# Cost benefit of investment on quality in pharmaceutical manufacturing: WHO GMP pre- and post-certification of a Nigerian pharmaceutical manufacturer

**DOI:** 10.1186/s12913-017-2610-8

**Published:** 2017-09-18

**Authors:** Chimezie Anyakora, Obinna Ekwunife, Faith Alozie, Mopa Esuga, Jonathan Ukwuru, Steve Onya, Jude Nwokike

**Affiliations:** 1 Promoting the Quality of Medicines Program, U.S. Pharmacopeial Convention, Rockville, MD USA; 2 Department of Clinical Pharmacy and Pharmacy Management, Nnamdi Azikiwe University, Awka, Nigeria; 3 The Centre for Applied Research on Separation Science Lagos, Lagos, Nigeria; 4 Chi Pharmaceuticals Limited, Lagos, Nigeria

**Keywords:** Africa, Cost-benefit analysis, Good manufacturing practice, Nigeria, Pharmaceutical companies, Prequalification

## Abstract

**Background:**

Pharmaceutical companies in Africa need to invest in both facilities and quality management systems to achieve good manufacturing practice (GMP) compliance. Compliance to international GMP standards is important to the attainment of World Health Organization (WHO) prequalification. However, most of the local pharmaceutical manufacturing companies may be deterred from investing in quality because of many reasons, ranging from financial constraints to technical capacity. This paper primarily evaluates benefits against the cost of investing in GMP, using a Nigerian pharmaceutical company, Chi Pharmaceuticals Limited, as a case study. This paper also discusses how to drive more local manufacturers to invest in quality to attain GMP compliance; and proffers practical recommendations for local manufacturers who would want to invest in quality to meet ethical and regulatory obligations.

**Method:**

The cost benefit of improving the quality of Chi Pharmaceuticals Limited’s facilities and system to attain WHO GMP certification for the production of zinc sulfate 20-mg dispersible tablets was calculated by dividing the annual benefits derived from quality improvement interventions by the annual costs of implementing quality improvement interventions, referred to as a benefit-cost ratio (BCR).

**Result:**

Cost benefit of obtaining WHO GMP certification for the production of zinc sulfate 20-mg dispersible tablets was 5.3 (95% confidence interval of 5.0–5.5).

**Conclusion:**

Investment in quality improvement intervention is cost-beneficial for local manufacturing companies. Governments and regulators in African countries should support pharmaceutical companies striving to invest in quality. Collaboration of local manufacturing companies with global companies will further improve quality. Local pharmaceutical companies should be encouraged to key into development opportunities available for pharmaceutical companies in Africa.

## Background

The African continent has poor health indicators compared to other continents, largely because the governments of African countries have not fully addressed health challenges facing their populace. One such health challenge is the lack of access to quality medicines by the populace, particularly those living in rural areas [1, 2], which in turn is caused by the small size of local pharmaceutical industry [3]. High dependence on imported medicines (estimated to be about 79%) is as a result of its weak pharmaceutical industry. Importation of medicines increases the cost of health care and also may result in an interrupted supply of medicines [4].

In 2015, more than 190 world leaders committed to 17 sustainable development goals (SDGs) to help end extreme poverty, fight inequality and injustice, and fix climate change. One of the targets of the third goal of SDGs is to achieve universal health coverage, including financial risk protection; access to quality essential health care services; and access to safe, effective, quality, and affordable essential medicines and vaccines for everyone [5]. Medicines or pharmaceuticals play an important role in the health care sector [6]. They are needed for prognosis, diagnosis, prevention, treatment of diseases, and even maintenance of health status. Notably, the quality of pharmaceuticals is at the core of the World Health Organization (WHO) constitution as health care systems are compromised by the availability of substandard drugs [7]. Pharmaceutical manufacturers are responsible for ensuring that medicines produced are of quality and fit for purpose [8] and for use by the general public [9]. The consequences of poor quality drugs include an increase in deaths and morbidity, increased adverse drug reactions (ADRs), and the development of drug resistance. It also reduces confidence of patients on the health care system [10].

Overreliance on donor funds for provision of health care and pharmaceutical products in Africa is not sustainable [2, 9, 11]. There is high demand for the supply of medicines in Africa. As an illustration, Africa is home to 75% of HIV cases and 90% of malaria deaths [12, 13]. Thus, the continent needs local medicine production to ensure continuous supply of medicines capable of handling the health challenges facing Africa rather than rely on external sources, which may disrupt supply and possibly increase the cost of health care provision. In addition to increasing availability and affordability of medicines, production of quality drugs would lead to reduced infiltration of substandard medicines into the market [14].

It is with this in mind that local pharmaceutical companies are viewed as being important in bringing sustainable solutions to the health problems in Africa [2, 11]. Local pharmaceutical companies in Africa must evolve in their capacity to provide high-quality pharmaceutical products to meet the growing health care need of the continent. To facilitate and maintain quality and safety of medicines in Africa, local production should be compliant with good manufacturing practices (GMPs). Manufacturers’ compliance to quality standard and national regulatory authorities is a shared goal of health and industrial policies [15]. GMPs, when adhered to, provide guidelines and methods that ensure quality is built into product and production processes [16]. GMPs address, among other elements, plant design, validated processes, and quality control of production cycles [11]. Regulatory agencies within countries also influence the availability or lack thereof of quality drugs manufactured by local manufacturing companies. For instance, in Nigeria the National Agency for Food and Drug Administration (NAFDAC) established in the early 1990s has reduced the proliferation of substandard pharmaceuticals in the country [14].

The majority of the pharmaceutical companies in Africa need to invest in quality-improving intervention (both facility- and system-based interventions), although implementation and inclusion has to be done efficiently to avoid wasteful use of resources [17]. With regard to facility-based interventions, the design of a pharmaceutical company should technically consider operations of the manufacturing plant [7, 8]. Elements such as heating ventilation and air conditioning (HVAC) system need to be built in to ensure quality in production [7]. Equipment should be suited to the operations and designed in ways that minimize risk [15]. Quality control and monitoring systems are more quality interventions that ensure optimal standard performance of companies, including their staff and products [6, 8]. Monitoring fosters identification, investigation, and prevention of deviations [8].

In relation to system-based interventions, quality control and monitoring systems are enhanced by the available information technology systems, which facilitates company’s operations and assessment of productivity [17]. Through this system, the verification and validation of the capability of the products produced or operational processes in a company is efficiently achievable through the use of collated data [6]. Defaults are identified easily and tackled. Besides surveillance purposes, control and monitoring systems should make allowances for detailing complaints [7]. An appropriate corrective and preventive action (CAPA) system should be ingrained in the company’s quality system to facilitate correction of emerging non-compliance issues. These issues vary and may include recalls, deviations, non-conformance, and product rejection. Investigations should be aimed at identifying the root cause of the issue arising. At the end of a CAPA review, the product and process in question should be improved and enhanced [8]. In addition, pharmaceutical manufacturing companies should engage in routine self-inspection. The personnel carrying out this inspection should be objective. In the case of recalls or deviations, self-inspection may also be warranted. The results of such an inspection should be analysed, solutions should be proffered, and implementation of necessary actions should be monitored [8]. However, carrying out an external audit may be more substantial instead of a self-inspection. External auditing ensures quality compliance in a company, because inefficient record keeping and poor auditing systems have an effect on organization’s output [7, 11]. External auditing can be done within the company or it can be extended to include suppliers and other arms involved in distribution to consumers. The end game of auditing is improvement of the pharmaceutical processes and products [7, 8].

WHO supports the development of local manufacturing companies in developing countries and, in particular, Africa. WHO GMP compliance is important for attaining of WHO prequalification [15]. WHO prequalification offers great opportunity for local West African pharmaceutical companies being able to distribute locally manufactured drugs to other Economic Community of West African States (ECOWAS) countries through the application for international medicines tender [15]. Unfortunately, none of the medicines produced currently by pharmaceutical manufacturers in West Africa has achieved WHO prequalification [15]. Governments of West African countries should urge their local pharmaceutical manufacturing companies to apply for WHO prequalification [4]. Most of the local pharmaceutical manufacturing companies may be deterred from investing in quality because of many reasons, ranging from financial constraints to technical capacity. An appraisal of a local West African company that has attained WHO GMP certification or, better still, WHO prequalification can act as an incentive to other local manufacturers. It is also important to examine how drug regulatory agencies of different West African governments could drive local manufacturers to invest in quality to attain GMP compliance and adhere to ethical and regulatory obligations.

This paper primarily evaluates benefit against the cost of investing in quality to attain GMP compliance using a Nigerian pharmaceutical company, Chi Pharmaceuticals Limited, as a case study. This paper also discusses how to drive more local manufacturers to invest in GMP compliance and proffers practical guidance to local manufacturers who would want to make investments in quality to meet ethical and regulatory obligations.

## Method

### Study design

This study used a quasi-experimental design involving pre- and post-assessment of Chi Pharmaceuticals Limited [18]. The study estimated the resources used to improve quality of Chi Pharmaceuticals Limited’s facilities and system so as to attain WHO GMP certification for the production of zinc sulfate 20-mg dispersible tablets--a project that lasted for 4 years (2011–2015). Chi Pharmaceutical Limited is a relatively large organization with a staff of 131 and a range of products totalling 30. The line of products manufactured by Chi Pharmaceutical Limited include those that are analgesic, antidiarrheal, anthelmintic, antimalarial, antibacterial, and antipsychotic. Chi Pharmaceutical Limited’s manufacturing facility was certified WHO GMP compliant in 2014. The company produces zinc sulfate in this facility. Zinc sulphate is used in the treatment of diarrhea in children. This drug is of interest to Nigeria because diarrhea is the second leading cause of death of children under 5 years of age in the country, accounting for 11% of their mortality rate [19].

Benefits resulting from Chi Pharmaceutical Limited’s investment in quality in the year following the completion of investment to attain WHO GMP certification for zinc sulfate 20-mg dispersible tablets (i.e., in 2016) were also assessed. Finally, the cost benefit of quality improvement investment was calculated by dividing annual benefits resulting from investment in quality by the annual costs of investment in quality.

### Study instrument and data collection

An in-depth interview guide was developed by the research team in consultation with industrial experts. As shown in Table 1, the guide included items relating to resources utilized in improving quality as well as possible benefits that could result in investment in quality. Using the developed guide, an in-depth interview was performed with the managing director of Chi Pharmaceuticals Limited, Dr. Steve Onya, on 12 August 2016. The interview lasted for 3 h and was conducted by a four-man research team. One member of the research team was the major interviewer while the other three members took notes during the interview. Notes were transcribed independently and then the transcribed data were cross-checked for consistency. The managing director of Chi Pharmaceuticals Limited was contacted for clarification in cases of data inconsistencies.

**Table 1:** Resource use items in the in-depth interview guide

Item	Description	Possible data source
A. Quality Improvement Interventions		
Capacity building of personnel	Cost of capacity building/training for personnel	Financial records/interview/training and records
Engagement of consultant/expert (e.g. validation/qualification of equipment, design of facility)	Consultancy fees and calibration cost	Financial records
Recruitment of additional staff	Staff cost	Financial records
Recalls	Logistics cost (cost of retrieval from market, litigation/penalties)	Interview
Audit	Cost of carrying out audit	Financial records/interview
Post-marketing surveillance (PMS)	Surveillance cost/cost of analysis	Interview
Preventive maintenance	Cost of spares, contractor or service provider	Financial records
Supply chain system (e.g., warehousing, delivery, vehicles)	1. Cost of setting up or upgrading warehouse	1. Financial records or estimate from a quantity surveyor
	2. Cost of maintaining quality storage or distribution of specialized products or materials	2. Financial records
Product development	Cost of product development, stability chamber etc.	Financial records/interview
Upgrade of facilities or acquiring new building/structures (e.g., building, HVAC, warehousing, equipment, analytical equipment, process, packaging)	Cost of upgrade or new acquisition	Financial records or estimates from a quantity surveyor/supplier
B. Benefits of Quality Improvement		
Sales volume	Unit sold, monetary income from sale measure pre and post implementation	Financial records
New business	Income from new business/customers	Sales records/financial records
Internal failures including rework, out-of-specification (OOS),batch records, errors, omissions	Cost of man-hours, machine hour/operation cost, material/product cost associated with internal failures	Factory/laboratory logs and interview
External failures including customer complaints, recalls, litigation, compliance directive by regulatory agency, cost of repeated inspections due to regulator’s quality concerns	1. Cost of product replacement, man-hour spent on investigation and cost 2. Logistic cost, cost of lost products, cost of destruction, man-hour in collation and compilation of reports 3. Potential litigation charges 4. Default fees, fines	Financial records, factory logs, interview and standard regulatory agency default fees
Potential new business	Potential to access new markets e.g. donor agencies, Ministry of Health (MOH), etc.	Willingness-to-pay by donor agency, MOH, etc.

### Resource use estimation

Resource use was estimated from the pharmaceutical company’s perspective. An activity-based costing approach was used to estimate the resources used to improve quality of Chi Pharmaceuticals Limited’s facilities and system to attain WHO GMP certification for the production of zinc sulfate 20-mg dispersible tablets. The cost of different items categorized as resource use was obtained mainly from an interview with the managing director of Chi Pharmaceutical Limited. The market prices of some of the items also were cross-checked to corroborate the data obtained during the interview.

Each cost item was valued from the base year of 2016 and represented as annual cost. All capital and equipment costs were annuitized with the assumption of 5 years as the lifetime of the equipment and 3% as the discount rate. Cost of buildings was annuitized with the assumption of 30 years as the lifetime of the building and 3% as the discount rate. All costs related to product development were annuitized with the assumption of 10 years as the lifetime of the product and 3% as the discount rate. The following annuity formula was used:

$$ Annual\kern0.5em value=\frac{PV}{\frac{1-{\left(1+r\right)}^{-n}}{r}} $$

where PV is present value, *r* is discount rate and *n* is number of life years.

Variability in cost items were captured using triangular distribution. For cost items with no information about their variance, a simple assumption was made that the standard error of the costs is equal to the estimate. Thus, this allowed gamma distribution to be fitted to the cost data using the method of moments approach.

### Benefit of quality investment

Benefit of quality improvement interventions was measured as revenue accruing from sales after implementation of quality improvement interventions. Specifically, annual sales volume of zinc sulfate 20-mg dispersible tablets in 2016 was assessed.

### Cost-benefit analysis

The cost benefit of improving quality of Chi Pharmaceuticals Limited’s facilities and system to attain WHO GMP certification for the production of zinc sulfate 20-mg dispersible tablets was calculated by dividing the annual benefits of quality improvement interventions from the annual costs of quality improvement interventions, referred to as a benefit–cost ratio (BCR) [20]. A ratio greater than 1 demonstrates a positive return on investment. Probabilistic sensitivity analysis (PSA) approach was used to assess parameter uncertainty. The PSA allowed for exploration of the joint uncertainty in benefits and costs across study parameters. Appropriate distributions of each uncertain parameter (e.g., gamma, triangular) were used for PSA calculation. A thousand Monte Carlo simulations were run and the mean (95% confidence interval) of BCR was presented. Specifically, a point estimate was drawn randomly from the distribution of each parameter used in estimating BCR. This was repeated 1000 times (1000 iterations) and then the average of the 1000 iterations with a 95% confidence interval was calculated. Monte Carlo simulation was conducted using Microsoft Excel 2010.

## Results

Resources used to improve quality of Chi Pharmaceuticals Limited’s facilities and system to attain WHO GMP certification for the production of zinc sulfate 20-mg dispersible tablets were categorized into trainings, analyses, engagement of consultants, staff recruitment, financial records, preventive maintenance, product development, and facility upgrades. Staff recruitment followed by facility upgrades consumed the most resources. Table 2 enumerates all the different items in each cost category.

**Table 2:** Annual resource use and benefit

Item^a^	Annual cost (U.S. dollars)	Minimum –Maximum or α/β	Distribution
Cost of investment in quality			
1. Cost of trainings			
Cost of training on product development	8206	7620–9792	Triangular
Cost of attending Joint UNICEF, UNFPA & WHO meeting with manufacturers and suppliers of diagnostic products, vaccines, finished pharmaceutical products and active pharmaceutical ingredients	6040	3020–9060	Triangular
Cost of attending local conference on WHO GMP	12	1/12	Gamma
Cost of internal GMP training	23,116	1/23,116	Gamma
Cost of PMG-MAN/NAFDAC training	8040	1/8040	Gamma
Cost of other external trainings (WAHO, WAPMAM, Cepat)	3157	1/3157	Gamma
2. Cost of analyses			
Cost of extra analysis in Nigeria	118	1/118	Gamma
Cost of extra analysis in Abroad	412	1/412	Gamma
3. Engagement of consultants			
Cost for calibration, validation of HVAC and qualification	45,226	41,608–52,613	Triangular
4. Staff recruitment			
Cost of employing eight staff including GMP trainer	125,000	96,016–112,500	Triangular
5. Financial records			
Cost of auditing international supplier	2352	1/2352	Gamma
Cost of auditing Nigeria supplier	450	1/450	Gamma
6. Preventive maintenance			
Maintenance cost (including spares, cost of hiring contractor)	15,285	7642–15,285	Triangular
7. Product development			
Cost of product development	14,654	1/14,654	Gamma
Cost of stability chamber	6551	5459–8734	Triangular
Design of palatability study	821	586–1172	Triangular
Palatability and adherence study	4689	1/4689	Gamma
Honorarium for key staff	352	1/352	Gamma
Time in analysis	5862	1/5862	Gamma
Cost of courier services	293	1/293	Gamma
8. Facility upgrades or new buildings/structures			
Equipment cost (HVAC, automated compression machine, blistering machine, HPLC, customized flame photometer, etc.)	92,801	83,521–102,081	Triangular
Cost of new building/upgrade (factory, laboratory)	357,135	321,421–357,135	Triangular
Cost of power generation	331,658	1/331,658	Gamma
Cost of change of filter	7642	1/7642	Gamma
Cost of change to more efficient blistering machine	38,212	1/38,212	Gamma
Cost of change to more efficient HPLC and Flame photometer	43,671	1/43,671	Gamma
Total	*1,141,753*		
Benefit of Quality Improvement			
Revenue from sales of zinc sulfate 20-mg dispersible tablets in 2016	6,500,000	1/6,500,000	Gamma
Total	6,500,000		

^a^All cost were annuitized or expressed as annual cost

Revenue accruing from the sale of zinc sulfate 20-mg dispersible tablets at the time of the interview in 2016 was $6.5 million (U.S.). The company projected revenue of $10 million by the end of 2016. Therefore, the cost benefit at the time of the interview for obtaining WHO GMP certification for the production of zinc sulfate 20-mg dispersible tablets was 5.3 (95% confidence interval of 5.0–5.5), while the cost benefit based on projected revenue will be 8.5 (95% confidence interval of 8.3–8.7).

## Discussion

This paper primarily evaluates the cost benefit of a local pharmaceutical manufacturing company (CHI Pharmaceuticals Limited) investing to attain WHO GMP certification. WHO GMP compliance is important to attaining WHO prequalification. To be WHO prequalified means that medicines produced by manufacturers have to be of an acceptable global quality, safety, and efficacy standard [15]. In addition, GMP compliance especially minimizes and manages pharmaceutical manufacturing risks.

Our evaluation showed that investment in quality is cost-beneficial. Based on our assessment of Chi Pharmaceutical Limited, the return on investment to improve quality to attain WHO GMP certification was about 5 times if the company did not. The results, however, have to be interpreted with the possible limitation of drawing our conclusions from examining only one company, which could cause generalization of our findings. However, this is unavoidable because Chi Pharmaceutical Limited is the only company in Nigeria that has advanced the most with GMP implementation.

Our findings highlight the enormous benefit for investment in quality in the pharmaceutical sector. GMP-compliant or prequalified local pharmaceutical manufacturing companies have the following advantages: eligibility for international, donor-sponsored tenders for medicines; improved capacity to manufacture products for entry into stringently regulated markets; increased potential to compete successfully for contract manufacture for local markets; faster registration; improved image and brand; reduced manufacturing costs due to improved capacity utilization and lower commercial operating costs; and increased capacity and skills to ensure quality manufacture across range of products [21]. As we discovered from the in-depth interview with the Chi Pharmaceutical Limited managing director, the company has obtained many positive prospects since acquiring WHO GMP compliance status. For instance, Chi Pharmaceutical Limited has already been approached by organizations such as USAID for the production of drugs used to prevent opportunistic infections (OIs) in HIV/AIDS patients. The Global Fund and other similar agencies have made similar requests to Chi Pharmaceutical Limited as a result of their quality culture. In addition, multinational companies have sought partnerships with Chi Pharmaceutical Limited because they typically search for low- and middle-income economies where the manufacture of products can be carried out at a reasonably low cost [6]. As determined from the interview with Chi Pharmaceutical Limited, the threshold for a profitable return on their investment in quality is 3 years. Because of donor-sponsored tenders for medicines won by the company, it is predicted that Chi Pharmaceutical Limited will yield a positive net gain in 2 years. This result, coupled with the Nigerian government’s efforts to support the local industry with priority to those with quality management systems in good standing, has motivated the company to increase its capacity. According to its managing director, Chi Pharmaceutical Limited will continue to invest in public health commodities as a differential business strategy. The company will not only reinvest profit, but it is willing to source additional funds to build capacity in both production and personnel to remain not only internally consistent to local regulatory requirements but externally competitive with global quality standards.

Based on the foregoing, it is necessary that other local manufacturers in Nigeria and West Africa are encouraged to rise to the occasion and take advantage of the numerous opportunities that abound. Expansion of production capacity in Nigeria and in other West African countries would give the country self-sufficiency in manufacturing essential drugs and also give national pharmaceutical industries the opportunity to compete globally [15]. Unfortunately, substantial financial resources are necessary for the development of sustainable processes and structures that ensure production of quality medicines [15]. Also, other challenges and obstacles affect investment in facility- and system-based quality-improving interventions in pharmaceutical industries, especially in sub-Saharan African countries. For Nigeria, particular obstacles encountered by manufacturers include high production cost, poor infrastructure, maintenance of equipment, lack of spare parts, outdated technology, and high interest rates [2, 8]. Operational activities of manufacturing companies are made cumbersome by the additional cost that has to be used in running generators and constructing water treatment facilities. These additional costs account for 25%–40% of production cost [2]. Political instability is another issue in Africa. Regulatory organizations require political backing to efficiently carry out their duties [6]. In addition, economic instability affects the willingness of private investors to invest in certain countries [9]. Another major obstacle is the time and possible financial loss as a result of closing operations to allow installation or upgrades to be executed. The HVAC system installation, for example, would require a shutdown period of 6 months to prevent cross-contamination [6]. Financial resources are necessary for development and for obtaining sustainable processes and equipment that ensure quality-assured medicines are produced. Equipment should be suited to the operations and should be designed in ways that minimize risk [15]. Efficient facilities and equipment, though, are in themselves not sufficient; highly skilled personnel are an integral part of GMPs because they are needed for the efficient running of pharmaceutical industries [6, 8, 11]. Unfortunately human resources are lacking in Africa. Companies need to continuously train their staff and engage qualified consultants [8].

Despite these challenges, the benefit of local pharmaceutical manufacturing industries investing in quality outweighs the challenges. Thus, local pharmaceutical manufacturing companies should be encouraged to make the investment to achieve GMP compliance. The following sections discuss how to drive more local manufacturers to invest in GMP and proffer practical guidelines for local manufacturers who would want to invest in quality to meet ethical and regulatory obligations. Particular emphasis is placed on the Nigerian pharmaceutical sector; however, these suggestions are applicable to other pharmaceutical companies in sub-Saharan African countries.

### Role of government

Government can play a role in increasing the capacity of local pharmaceutical companies to manufacture quality medicines. Companies that have already invested or are investing in improving quality in their pharmaceutical manufacturing would need support from regulators and government of the country (UNIDO, 2011). Local manufacturing companies may be deterred by the cost required to reach and operate at an international quality standard, such as the standard required for WHO prequalification [22]. Capital needed for manufacturers in Africa runs into the millions of dollars and may require long-term financing. Most pharmaceutical companies in Africa are limited in their ability to upgrade because of a lack of access to financing [11]. Governments of developing countries can make available grants, soft loans, and subsidies and improve financing of health services such that they are able to patronize local manufacturers, facilitate joint ventures, and encourage international cooperation [23].

Also, a dichotomy often exists between GMP standards and the national regulatory requirements in African countries. This fact was noted by the managing director of Chi Pharmaceutical Limited during our in-depth interview with him. The implication is that some local manufacturers do not see the need to upgrade because there is no incentive. Therefore, it is necessary that national regulatory requirements are aligned with GMP standards. Also provisions should be made for the better recognition of companies that are WHO prequalified or WHO GMP certified. For instance, the government can award certain contracts to WHO GMP-compliant companies. Lastly, regulators and government would have to ensure that companies producing substandard drugs are not allowed to remain in circulation [2].

### Training

Local manufacturing companies in Nigeria currently satisfy only 25% of local demand [2]. This speaks to the need to increase the capacity of local pharmaceutical companies to manufacture quality medicines. Although local pharmaceutical production strengthening would take time, certain interventions would help fast track results. One such intervention is the inclusion of industrial training in the module for pharmaceutical schools in Nigeria and West Africa at large. To achieve that goal, industrial training should be done in GMP-compliant companies. Such trainings would improve the sustainability of the processes and quality of new pharmaceutical industries [11].

### Collaboration

Local manufacturing companies considered to be operating at a good or stable GMP level should begin to explore working with global companies either by joint venture or licensing agreement to further improve their quality [6]. For instance, a few companies in Nigeria have keyed into collaboration with foreign companies. Chi Pharmaceuticals Limited is currently developing drugs for the prevention HIV in collaboration with foreign companies. Such opportunities will increase revenue for Chi Pharmaceuticals Limited, lead to job creation, and could lead to foreign exchange savings. This is possible because of the effort of Chi Pharmaceutical Limited to improve the quality of its manufacturing.

### Foreign aid

Local pharmaceutical industries are also encouraged to key into development opportunities available for pharmaceutical companies in Africa. For instance, the African Development Bank has plans in place to support pharmaceutical industries. These plans include capacity building, learning events such as visitation of policy makers and industrialists to India, and setting up dialogues between public and private sector to discuss opportunities and challenges. Capacity building also is made available by other organizations such as the USAID-funded Promoting the Quality of Medicines (PQM) program, implemented by the U.S. Pharmacopeial Convention and WHO. The activities of the WHO prequalification team include, among others, capacity building of regulators and provision of guidance to manufacturers [15].

## Conclusion

This study shows that it is cost-beneficial for local manufacturing companies to invest in quality improvement interventions. Investment made to improve quality also has enormous benefit to the countries where the local manufacturing companies are located because of substantial foreign exchange savings and job creation. It is therefore imperative that governments and regulators in African countries support pharmaceutical companies striving to invest or that have already invested in improving their quality. Further, adapting the curriculum of pharmacy schools and chemistry programs in order to create a pipeline of quality assurance professionals will improve the sustainability of the processes and quality of new pharmaceutical companies. Collaboration of local manufacturing companies with global companies will further improve the former’s quality. Local pharmaceutical companies should be encouraged to key into development opportunities available for pharmaceutical companies in Africa.

## Abbreviations

ADR: Adverse drug reaction
AIDS: Acquired Immune Deficiency Syndrome
BCR: Benefit cost ratio
CAPA: Corrective and preventive action system
CBA: Cost-benefit analysis
ECOWAS: Economic Community of West African States
GMP: Good Manufacturing Practice
HIV: Human immunodeficiency virus
HVAC: Heating, ventilation and air conditioning
MOH: Ministry of Health
NAFDAC: National Agency for Food and Drug Administration and Control
OOS: Out of specification
PMS: Post-marketing surveillance
PSA: Probabilistic sensitivity analysis
SDG: Sustainable Development Goal
WHO: World Health Organization
